# Urinary genome detection and tracking of Hantaan virus from hemorrhagic fever with renal syndrome patients using multiplex PCR-based next-generation sequencing

**DOI:** 10.1371/journal.pntd.0009707

**Published:** 2021-09-28

**Authors:** Seungchan Cho, Won-Keun Kim, Jin Sun No, Seung-Ho Lee, Jaehun Jung, Yongjin Yi, Hayne Cho Park, Geum-Young Lee, Kyungmin Park, Jeong-Ah Kim, Jongwoo Kim, Jingyeong Lee, Daesang Lee, Dong Hyun Song, Se Hun Gu, Seong Tae Jeong, Jin-Won Song

**Affiliations:** 1 Department of Microbiology, Korea University College of Medicine, Seoul, Republic of Korea; 2 Department of Microbiology, College of Medicine, Hallym University, Chuncheon, Republic of Korea; 3 Institute of Medical Science, College of Medicine, Hallym University, Chuncheon, Republic of Korea; 4 Division of High-risk Pathogens, Bureau of Infectious Diseases Diagnosis Control, Korea Disease Control and Prevention Agency, Cheongju, Republic of Korea; 5 Department of Preventive Medicine, Gachon University College of Medicine, Incheon, Republic of Korea; 6 Division of Nephrology, Department of Internal Medicine, Dankook University Hospital, Cheonan, Republic of Korea; 7 Department of Internal Medicine, Kangnam Sacred Heart Hospital, Seoul, Republic of Korea; 8 BK21 Graduate Program, Department of Biomedical Sciences, Korea University College of Medicine, Seoul, Republic of Korea; 9 4th R&D Institute, Agency for Defense Development, Daejeon, Republic of Korea; NIAID Integrated Research Facility, UNITED STATES

## Abstract

**Background:**

Hantavirus infection occurs through the inhalation of aerosolized excreta, including urine, feces, and saliva of infected rodents. The presence of Hantaan virus (HTNV) RNA or infectious particles in urine specimens of patient with hemorrhagic fever with renal syndrome (HFRS) remains to be investigated.

**Methodology/Principal findings:**

We collected four urine and serum specimens of Republic of Korea Army (ROKA) patients with HFRS. We performed multiplex PCR-based next-generation sequencing (NGS) to obtain the genome sequences of clinical HTNV in urine specimens containing ultra-low amounts of viral genomes. The epidemiological and phylogenetic analyses of HTNV demonstrated geographically homogenous clustering with those in *Apodemus agrarius* captured in highly endemic areas, indicating that phylogeographic tracing of HTNV genomes reveals the potential infection sites of patients with HFRS. Genetic exchange analyses showed a genetic configuration compatible with HTNV L segment exchange in nature.

**Conclusion/Significance:**

Our results suggest that whole or partial genome sequences of HTNV from the urine enabled to track the putative infection sites of patients with HFRS by phylogeographically linking to the zoonotic HTNV from the reservoir host captured at endemic regions. This report raises awareness among physicians for the presence of HTNV in the urine of patients with HFRS.

## Author summary

Hantavirus transmission to humans occurs via inhalation of aerosolized excreta, including urine, feces, and saliva of infected rodents. Currently, no report for the etiological evidence associated with urinary Hantaan virus (HTNV) from patients with hemorrhagic fever with renal syndrome (HFRS) is available. Here, we conducted multiplex PCR-based next-generation sequencing (NGS) using urine and serum specimens from four Republic of Korea Army (ROKA) patients with HFRS. The epidemiological and phylogenetic analyses using whole or partial genome sequences of HTNV from urine and serum demonstrated homogenous genetic clustering with HTNV from clinical specimens, circulating at highly endemic sites of patient infection. Among the sequences from ROKA patients, the genomic configuration of ROKA16-10 demonstrated occurrences of the genetic reassortment. Our results suggest that whole or partial genome sequences of HTNV from the urine enabled to track the putative infection sites of patients with HFRS by phylogeographically linking to the zoonotic HTNV from the reservoir host captured at endemic regions. This result provides new insights into presence of HTNV in the urine of patients with HFRS among physicians.

## Introduction

Hantaviruses (genus *Orthohantavirus*, family *Hantaviridae*, and order *Bunyavirales*) are enveloped negative-sense, single-stranded tripartite RNA viruses, consisting of large (L), medium (M), and small (S) segments [1]. Hantavirus infections cause hemorrhagic fever with renal syndrome (HFRS) and hantavirus cardiopulmonary syndrome in humans. *Hantaan orthohantavirus* (Hantaan virus, HTNV) and *Seoul orthohantavirus* infection present 150,000 clinical cases annually, with fatality rates ranging from 1–15% [2]. Hantavirus transmission to humans occurs via inhalation of aerosolized excreta, including urine, feces, and saliva of infected rodents. A critical hallmark of HFRS is capillary leakage that results in edema and hemorrhage, suggesting that the vascular endothelium is damaged by a cytokine storm against hantavirus infection [3]. The clinical course of HFRS typically defines five phases: febrile, hypotensive, oliguric, diuretic, and convalescent [4]. Initially, HFRS represents a febrile phase characterized by fever, pain, and edema for three to five days. The hypotensive phase lasts a few hours, and is marked by internal bleeding, decreased blood pressure, proteinuria, and thrombocytopenia. The oliguric phase indicates renal dysfunction, hypervolemia, and blood electrolyte imbalance, lasting three to seven days. The diuretic phase is marked by increased urine output for two weeks. The convalescent phase is a recovery stage that lasts three to six months, and is marked by progressive improvements in glomerular filtration rate, renal blood flow, and urine output control.

Clinical HTNV RNA loads were observed in the plasma during the early stages of HFRS (febrile/hypotensive and oliguric phase; 12 days after the onset of disease) [5]. Reverse transcription polymerase chain reaction (RT-PCR) is a laboratory molecular diagnosis method for HTNV RNA detection in serum. The urinary viral loads of severe acute respiratory syndrome coronavirus 2 (SARS-CoV-2) were detected in Coronavirus disease 19 (COVID-19) patients, and the infectious virus particles were isolated from urine and respiratory specimens [6]. Urine samples of Zika virus (ZIKV)-infected patients were found to be positive by real-time RT-PCR [7]. In 2012, real-time RT-PCR was implemented for the detection of West Nile virus (WNV) RNA in urine samples during the outbreak [8]. *Puumala orthohantavirus* (PUUV) RNA was detected in the saliva of patients with mild HFRS [9]. *Andes orthohantavirus* (ANDV) was diagnosed using human apolipoprotein H-coated enzyme-linked immunosorbent assay in urine and serum samples [10]. However, the presence of HTNV RNA or infectious virus particles in HFRS urinary specimens remains to be investigated.

Reassortment and recombination are major molecular mechanisms for genetic exchanges that result in divergent virus progenies [11]. The reassortment and recombination of hantaviruses have been reported naturally and experimentally. The inter-lineage reassortments of PUUV were detected in northern Finland [12]. Genetic exchange of Sin Nombre virus (SNV) has been reported in nature and *in vitro* [13]. ANDV has high molecular diversity in the M segment, resulting in five diverged clades related to its geographic origins in South America [14]. Recently, we revealed natural occurrences and dynamic genetic exchanges of rodent- and shrew-borne hantaviruses [15,16].

In this study, multiplex PCR-based NGS facilitated urinary genomic acquisition of HTNV, revealing the putative infection sites of patients with HFRS and a genetic exchange event. This report provides insights into non-invasive diagnostics and genomic epidemiology of urinary HTNV genome sequencing.

## Results

### Epidemiologic and clinical characteristics of study cases

Patient Republic of Korea Army (ROKA)16-10 conducted military activity in Pocheon-si on November 21, 2016 (**Table 1**). The soldier exhibited clinical HFRS symptoms on December 12, 2016 and was admitted to the Armed Forces Capital Hospital (AFCH) in the Republic of Korea (ROK). He was diagnosed with HFRS with fever, chills, headache, conjunctival hemorrhage, and petechiae on December 14 (**Table 2**). ROKA16-10 had clinical outcomes with a minimum systolic blood pressure of ≤95 mmHg for 9 days, serum albumin of ≤3.0/dL for 5 days, platelet count of ≤89,000/μL for 3 days, urine volume of ≥3,500 mL/d for 7 days, and urine RBC detection (**Table 3**). Patient ROKA16-12, assigned to Yangju-si, had conducted military activity in Paju-si. The soldier exhibited the onset of clinical symptoms on December 13, 2016 and was admitted to AFCH with fever, headache, and conjunctival hemorrhage on December 19. WBC ≥14,000/μL for 4 days, a minimum systolic blood pressure ≤95 mmHg for 12 days and ≥141 mmHg for 9 days, serum albumin of ≤3.0/dL for 3 days, platelet count of ≤89,000/μL for 1 day, urine volume of ≥3,500 mL/d for 7 days, and urine RBC were observed in ROKA16-12. Patient ROKA17-2 had conducted military training in Paju-si on December 12–16, 2016. The soldier exhibited clinical symptoms on January 6, 2017. ROKA 17–2 was diagnosed with HFRS with fever and chills on January 10, 2017. Laboratory studies of ROKA17-2 indicated a minimum systolic blood pressure of ≤95 mmHg for 11 days and ≥141 mmHg for 10 days, platelet count of ≤89,000/μL for 1 day, and urine volume of ≥3,500 mL/d for 4 days. Patient ROKA17-15 conducted military training in Yeoncheon-gun on October 19, 2017. The soldier exhibited clinical HFRS symptoms on October 22, 2017. On October 27, the soldier was diagnosed with HFRS with fever, chills, headache, myalgia, and oliguria. ROKA17-15 had clinical outcomes with a minimum systolic blood pressure of ≤95 mmHg for 18 days and ≥141 mmHg for 3 days, platelet count of ≤89,000/μL for 3 days, urine volume of ≥3,500 mL/d for 5 days, and ≤500 mL/d for 1 day, and urine RBC detection.

**Table 1:** Summary of Republic of Korea Army (ROKA) with hemorrhagic fever with renal syndrome (HFRS) patient information.

Patient	Site	Onset	Date of diagnosis	Date of sample collected	Date of outdoor activities	Type	IFA for HTNV^a^	RT-PCR for HTNV^b^			Ct value of HTNV
								L segment (2,946–3,335)	M segment (1,970–2,342)	S segment (973–1,253)	
ROKA 16–10	Pocheon-si	Dec. 12, 2016	Dec. 14, 2016	Dec. 15, 2016	Nov. 21, 2016	Urine	<1:32	-	+	+	30.1
						Serum	<1:32	+	+	+	29.1
ROKA 16–12	Paju-si	Dec. 13, 2016	Dec. 18, 2016	Dec. 19, 2016	Nov. 14, 2016	Urine	1:128	+	+	-	40
						Serum	1:512	+	+	+	35.8
ROKA 17–2	Paju-si	Jan. 6, 2017	Jan. 10, 2017	Jan. 13, 2017	Dec. 12–16, 2016	Urine	1:512	-	+	-	40
				Jan. 11, 2017		Serum	1:512	+	+	+	35.4
ROKA 17–15	Yeoncheon-gun	Oct. 22, 2017	Oct. 27, 2017	Oct. 29, 2017	Oct. 19, 2017	Urine	1:128	-	+	+	40
				Oct. 27, 2017		Serum	1:512	+	+	+	31.3

IFA, indirect immunofluorescence antibody assay; RT-PCR, reverse transcription-polymerase chain reaction; pos, positive; neg, negative; HTNV, Hantaan virus; ROKA, Republic of Korea Army.

^a^Indirect immunofluorescence antibody test for anti-HTNV IgG antibody.

^b^RT-PCR was performed using HTNV L, M, and S segment-specific outer and inner primer sets (17).

**Table 2:** Epidemiologic and clinical characteristics of hemorrhagic fever with renal syndrome (HFRS) patients.

Patient	Site	Onset	Date of sample collected	Clinical symptoms									
				Fever	Chill	Headache	Myalgia	Conjunctival hemorrhage	Petechiae	Dyspnea	Hematuria	Polyuria	Oliguria
ROKA 16–10	Pocheon-si	Dec. 12, 2016	Dec. 15, 2016	39.2	+	+	-	+	+	-	-	-	-
ROKA 16–12	Paju-si	Dec. 13, 2016	Dec. 19, 2016	37.5	-	+	-	+	+	-	-	-	-
ROKA 17–2	Paju-si	Jan. 6, 2017	Jan. 11, 2017	38.0	+	-	-	-	-	-	-	-	-
			Jan. 13, 2017	37.4	-	-	-	-	-	-	-	-	-
ROKA 17–15	Yeoncheon-gun	Oct. 22, 2017	Oct. 27, 2017	38.1	+	+	+	+	-	-	-	-	+
			Oct. 29, 2017	37.5	-	-	-	+	-	-	-	-	+

ROKA, Republic of Korea Army

**Table 3:** Clinical outcomes and duration for hemorrhagic fever with renal syndrome (HFRS) patients.

Phase	Observation	ROKA16-10	ROKA16-12	ROKA17-2	ROKA17-15
	Severity	Moderate	Moderate	Mild	Moderate
**Febrile**	Temperature over 38 (≥6 days)	- (3 days)	-	- (1 day)	- (1 days)
	WBC (≥14000/μL)	-	+ (4 days)	-	-
**Hypotensive**	Min. systolic B.P. (≤95 mmHg)	+ (9 days)	+ (12 days)	+ (11 days)	+ (18 days)
	Serum albumin (≤3.0/dL)	+ (5 days)	+ (3 days)	-	-
	Min. platelets (≤89,000/μL)	+ (3 days)	+ (1 days)	+ (1 day)	+ (3 days)
	Urine RBC	+	+	-	+
**Oliguric and diuretic**	Max. systolic B.P. (≥141 mmHg)	-	+ (9 days)	+ (10 days)	+ (3 days)
	Max. urine volume (≥3,500 mL/d)	+ (7 days)	+ (7 days)	+ (4 days)	+ (5 days)
	Min. urine volume (≤ 500 mL/d)	-	-	-	+ (1 day)
	eGFR (≤15 mL/min)	-	-	ND	ND
**Event**	Transfusion	-	-	-	-
	Hemodialysis	-	-	-	-
**Total**		**5/12**	**6/12**	**4/12**	**6/12**

ND: Not determined; ROKA, Republic of Korea Army.

WBC, whole blood cell; B.P., blood pressure; RBC, red blood cell; eGFR, epidermal growth factor receptor.

### Laboratory diagnostics of HFRS cases

The clinical specimens were examined for anti-HTNV Immunoglobulin G (IgG) by indirect immunofluorescence antibody test (IFAT) **(Table 1)**. There was no detectable for anti-HTNV IgG in the urine and serum of ROKA16-10. However, the partial genome sequences of HTNV L, M, and S segments were obtained by RT-PCR in the serum. The urine specimen contained the genome sequences of HTNV M and S segments. Upon examination of the viral load in clinical specimens, the Ct value was 29.1 in serum and 30.1 in urine. Anti-HTNV IgG and HTNV RNA were detected in specimens (urine and serum) of patient ROKA16-12, ROKA17-2, and ROKA17-15. However, Ct value was undetectable in the urine samples.

### Whole-genome sequencing of HTNV using multiplex PCR-based NGS

To acquire the tripartite genome sequences of HTNV from patient specimens (urine and serum), we performed multiplex PCR-based NGS. On average, 240,792 (16.0%), 571,690 (37.8%), and 315,487 (20.8%) reads out of a total of 1,512,915 reads mapped to the reference sequence for the HTNV L, M, and S segments, respectively **(Table 4)**. Both the portion of mapped reads and the depth of coverage were lowest in the L segment. The whole-genome sequences of HTNV were recovered from the urine and serum of ROKA16-10. The coverage rates of HTNV tripartite genome sequences ranged from 97.6% to 99.6% in the clinical specimens. The genome sequences of the 3′ and 5′ ends were empirically determined due to the conserved region of the family *Hantaviridae* [17]. The partial genome sequences of HTNV from ROKA16-12, ROKA17-2, and ROKA17-15 were obtained from urine and serum. The S segments of ROKA16-12 and ROKA17-2 were fully recovered from the clinical specimens, while the L and M segments were not entirely obtained. In ROKA17-15, the L, M, and S segments of HTNV were partially acquired with low coverage rates.

**Table 4:** Summary of total reads and read mapping to Hantaan virus (HTNV) reference genome by HTNV multiplex polymerase chain reaction-based next generation sequencing.

Code No.	Type	Viral reads/ Total reads (%)	Total reads	L segment			M segment			S segment		
				Viral reads	Depth of reads	Coverage rate (%)	Viral reads	Depth of reads	Coverage rate (%)	Viral reads	Depth of reads	Coverage rate (%)
ROKA 16–10	Urine	92.9	1,279,702	416,963	9,573.6	97.6	360,795	14,966.6	99.6	410,584	36,313.4	99.2
	Serum	93.5	1,710,252	644,785	14,804.4	99.6	544,028	22,567.5	99.6	409,608	36,227.1	99.2
ROKA 16–12	Urine	38.6	1,641,756	11,492	263.8	91.5	610,836	25,338.8	94.5	10,725	948.5	99.2
	Serum	77.3	1,492,918	222,249	5,102.9	99.2	500,613	20,766.5	99.6	431,517	38,164.8	99.2
ROKA 17–2	Urine	60.1	627,968	28,610	656.8	76.4	345,136	14,317.0	98.6	3,652	322.9	99.2
	Serum	89.6	594,166	53,118	1,219.6	92.3	253,622	10,520.8	98.6	225,809	19,971.3	99.2
ROKA 17–15	Urine	50.5	2,287,976	30,810	707.4	68.2	1,030,365	42,741.9	79.6	94,965	8,399.0	79.8
	Serum	96.6	2,468,578	518,309	11,900.6	99.6	928,124	38,500.7	99.6	937,032	82,874.3	99.2
**Average**			**1,512,915**	**240,792**	**5,528**.**6**		**571,690**	**23,715**.**0**		**315,487**	**27,902**.**7**	

ROKA, Republic of Korea Army

### Phylogeographic and reassortment analyses of HTNV genomes from HFRS patients

**Fig 1:**
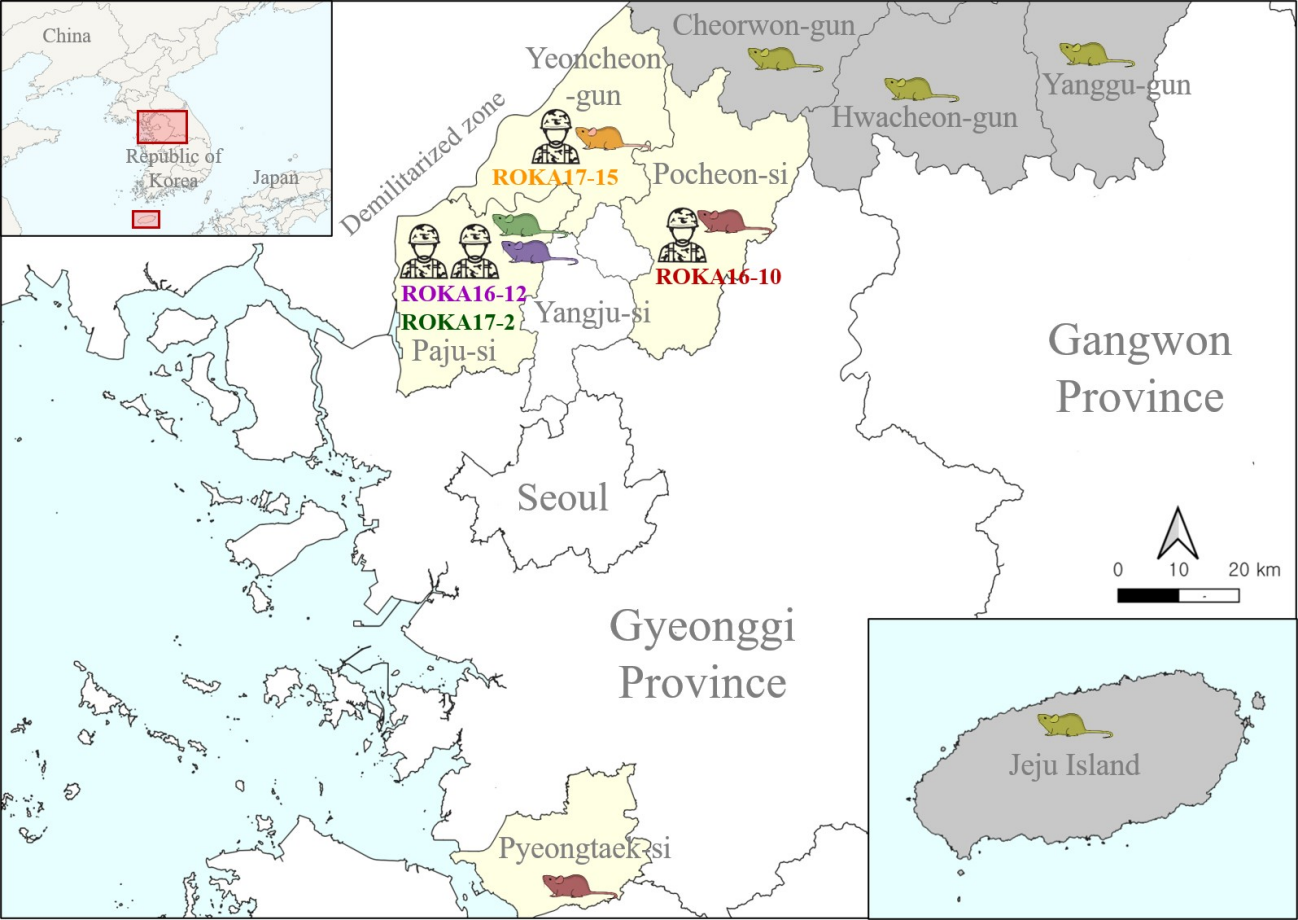
Geographic map of hemorrhagic fever with renal syndrome (HFRS) patients in this study. A geographic map shows the sites of patients with HFRS and rodents collected at highly endemic area in Gyeonggi and Gangwon Provinces. The red, purple, green, and orange color indicate trapped rodents which shows phylogeographic relation with each ROKA16-10, ROKA16-12, ROKA17-2, and ROKA17-15 patient, respectively. The yellow color indicates trapped rodents in Gangwon Province and Jeju Island. Paju-si, Yangju-si, Pocheon-si, Yeoncheon-gun, and Pyeongtaek-si are located in Gyeonggi Province, and Cheorwon-gun, Hwacheon-gun, and Yanggu-gun are located in Gangwon Province, Republic of Korea. We used the Quantum Geographical Information System (QGIS) software V.3.4.10. and Adobe Illustrator CS to create the geographic map.

**Fig 2:**
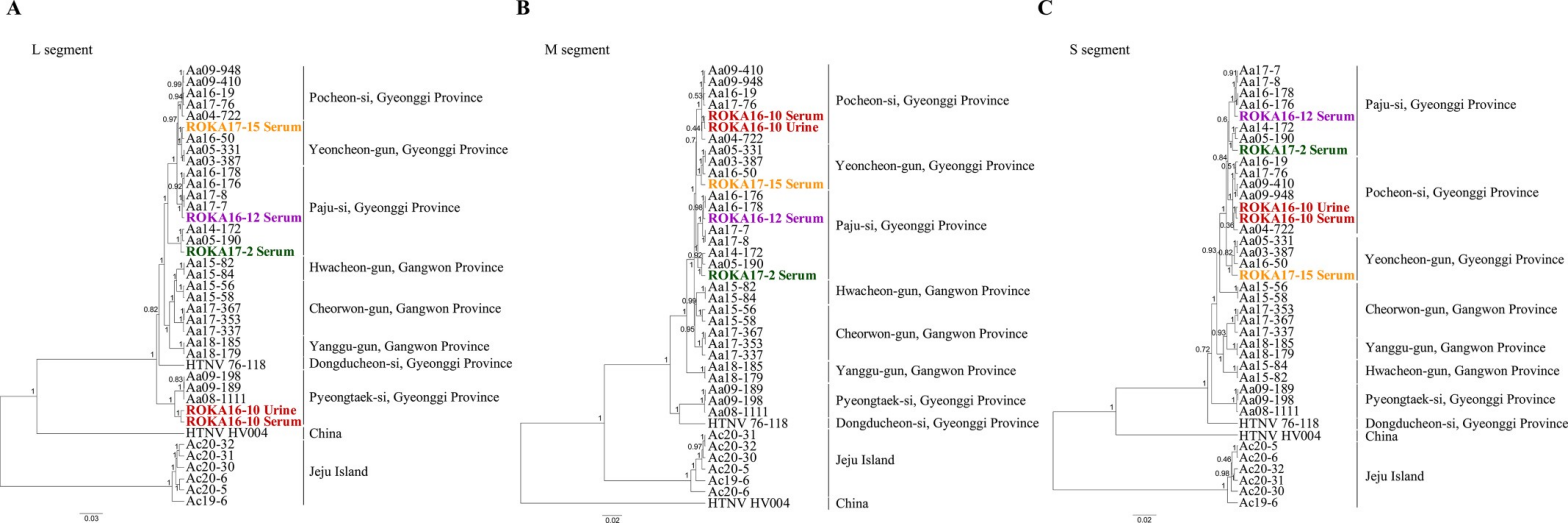
Phylogenetic analysis of whole-genome sequences of Hantaan virus (HTNV) L, M, and S segments from patients with hemorrhagic fever with renal syndrome (HFRS). Phylogenetic relationships of the whole-genome sequences of the HTNV L, M, and S segments were inferred using Bayesian inference in BEAST (v1.10.4), using the GTR+G+I, GTR+G, and TN93+G models based on 6,530 nt of the L segment (A), 3,616 nt of the M segment (B), and 1,696 nt of the S segment (C), respectively. After running the Markov chain Monte Carlo analyses until adequate sample sizes (ESS>200) were obtained, TreeAnnotator (v2.5.4) was used to summarize a maximum clade credibility tree from the posterior tree distribution, using a 25% burn-in. Numbers along branches are posterior probability. Posterior probabilities of 0 are induced by the rounding of values to 2 decimal places. The scale bar represents nucleotide substitutions per site. The following hantaviral sequences were included in the analysis: Aa03-387 (L segment, KT934958; M segment, KT934992; S segment, KT935026), Aa04-722 (L segment, KU2071740; M segment, KU207182; S segment, KU207190), Aa05-190 (L segment, KT934959; M segment, KT934993; S segment, KT935027), Aa05-331 (L segment, KT934962; M segment, KT934996; S segment, KT935030), Aa08-1111 (L segment, KY594712; M segment, KY594715; S segment, KY594718), Aa09-189 (L segment, KY594713; M segment, KY594716; S segment, KY594719), Aa09-198 (L segment, KY594714; M segment, KY594717; S segment, KY594720), Aa09-410 (L segment, KU207177; M segment, KU207185; S segment, KU207193), Aa09-948 (L segment, KT934966; M segment, KT935000; S segment, KT935034), Aa14-172 (L segment, KT934974; M segment, KT935008; S segment, KT935042), Aa15-56 (L segment, KU207179; M segment, KU207187; S segment, KU207195), Aa15-58 (L segment, KU207180; M segment, KU207188; S segment, KU207196), Aa15-82 (L segment, MT012572; M segment, MT012560; S segment, MT012548), Aa15-84 (L segment, MT012573; M segment, MT012561; S segment, MT012549), Aa16-19 (L segment, MK548664; M segment, MK548655; S segment, MK548646), Aa16-50 (L segment, MK548667; M segment, MK548658; S segment, MK548649), Aa16-176 (L segment, MH598473, M segment, MH598487, S segment, MH598501), Aa16-178 (L segment, MH598474, M segment, MH598488, S segment, MH598502), Aa17-7 (L segment, MH598475; M segment, MH598489; S segment, MH598503), Aa17-8 (L segment, MH598476; M segment, MH598490; S segment, MH598504), Aa17-76 (L segment, MK548672; M segment, MK548663; S segment, MK548654), Aa17-337 (L segment, MT012574; M segment, MT012562; S segment, MT012550), Aa17-353 (L segment, MT012575; M segment, MT012563; S segment, MT012551), Aa17-367 (L segment, MT012576; M segment, MT012564; S segment, MT012552), Aa18-179 (L segment, MT012580; M segment, MT012568; S segment, MT012556), Aa18-185 (L segment, MT012581; M segment, MT012569; S segment, MT012557), Ac19-6 (L segment, MW219756; M segment, MW219762; S segment, MW219768), Ac20-5 (L segment, MW219757; M segment, MW219763; S segment, MW219769), Ac20-6 (L segment, MW219758; M segment, MW219764; S segment, MW219770), Ac20-30 (L segment, MW219759; M segment, MW219765; S segment, MW219771), Ac20-31 (L segment, MW219760; M segment, MW219766; S segment, MW219772), Ac20-32 (L segment, MW219761; M segment, MW219767; S segment, MW219773), HTNV 76–118 (L segment, NC_005222; M segment, M14627; S segment, M14626), and HV004 (L segment, JQ083393; M segment, JQ083394; S segment, JQ093395).

**Fig 3:**
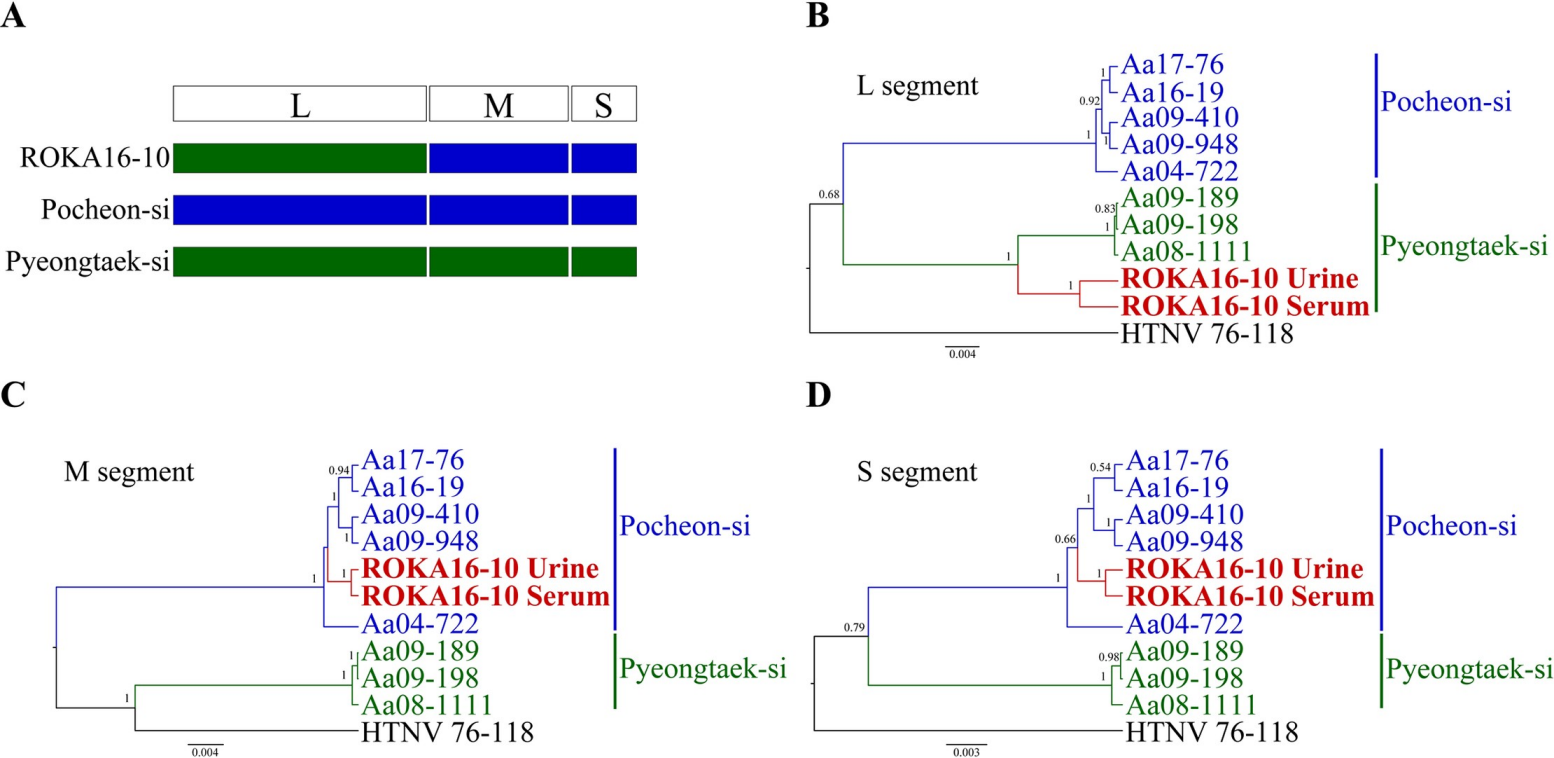
Evidence of a reassortment event in Hantaan virus (HTNV) on patient ROKA16-10 in Pocheon-si. (A) The Bootscan plot was based on a pairwise distance model using the RDP4 algorithm. Green and blue colors represent the comparison of ROKA16-10 (Pocheon-si) to Pyeongtaek-si and Pocheon-si groups, respectively. (B-D) Phylogenetic trees are shown for the L, M, and S segments of the reassortant ROKA16-10 using Bayesian inference in BEAST (v1.10.4).

The geographic location of patients with HFRS and rodents collected at highly endemic sites in Gyeonggi Province was described in **Fig 1**. The M and S segments of ROKA16-10 in the urine and serum formed a genetic lineage with HTNV identified in Pocheon-si **(Fig 2)**. However, the L segment of ROKA16-10 phylogenetically belonged to those of HTNV obtained in Pyeongtaek-si, Southern Gyeonggi Province **(Fig 3)**. To investigate the occurrence of genetic exchange events, the RDP recombination consensus score (RDPRCS) of ROKA16-10 was 0.678 with the P-value ranged from 1.495E-18 to 2.463E-37 in Recombination Detection Program 4 (RDP4). The Graph-incompatibility-based Reassortment Finder (GiRaF) analysis demonstrated that the L segment was a determinant for reassortment of HTNV ROKA16-10 with over 0.99 confidence levels. These results showed that ROKA16-10 may be a reassortant arising from the genetic exchange of the HTNV L segment between HTNV strains from Pocheon-si and Pyeongtaek-si. Phylogenetic analysis of partial genome sequences of ROKA16-12 revealed that the L, M, and S segments formed a homologous genetic group with HTNV acquired from *A*. *agrarius* collected in Paju-si. Phylogenetic analysis of ROKA17-2 and ROKA17-15 showed that the L, M, and S segments grouped with HTNV identified in Paju-si and Yeoncheon-gun, respectively. This phylogeographic tracking were well established with an epidemiological survey indicating that the patient worked at a possible exposed site.

## Discussion

Molecular diagnosis of urine and saliva samples has been explored for DNA and RNA virus infections. Urine samples seem suitable for the diagnosis of infection by severe fever with thrombocytopenia syndrome virus (SFTSV), SARS-CoV-2, hepatitis virus, WNV, ZIKV, and Dengue virus (DENV) [6–8,18–21]. PUUV RNA was detected in saliva and plasma from hospitalized nephropathia epidemica patients in northern Sweden [9]. In *Bunyavirales*, interhuman transmission was reported between households of ANDV in Argentina and Chile [22,23]. Dabie bandavirus from SFTSV-positive urine samples was transmitted to ferrets [24]. Although there is no report that human-to-human transmission of HTNV occurs through patient excreta, the risk of infection through urine or saliva still remains. In HTNV-positive *A*. *agrarius*, HTNV particles were observed in urine samples for up to 650 days [25]. HTNV antigens in urine sediments from patients with HFRS were detected from 7 to 40 days after onset [26]. The observation of HTNV particles in urine samples of patients with HFRS and rodents revealed the need for identification of the HTNV genome. In the present study, we demonstrated that whole-genome sequences of HTNV were recovered from urine and serum specimens of patients with HFRS. This is the first report of the identification of HTNV genome sequences found in the urine specimens. Further investigations are needed to evaluate whether infectious HTNV particles are excreted in urine, as has been identified for other arboviruses [8]. This aids in establishing a non-invasive diagnosis for the detection of HTNV RNA.

The indices of urinary acidification in patients with HFRS showed weak acidity (pH: 6.52±0.32 to 5.95±0.27) and a tendency to decrease as the phases progressed [27]. In addition, the urine of humans is a suitable environment for nucleic acid hydrolyzing enzymes caused by various ion concentrations and proper pH (from 5.0 to 7.0) [28]. Nucleic acid stabilizers (e.g., Buffer ATL, DNA/RNA Shield) improved the recovery of ZIKV, DENV, Venezuelan equine encephalitis virus, and Rift Valley fever virus RNA, particularly at low viral titers [29–32]. However, the stability of HTNV RNA in urine has not been reported yet. The whole-genome sequence of HTNV was recovered from the urine of ROKA16-10, but partial sequences of HTNV were obtained from three patients with HFRS with low coverage rate and depth of coverage. Several factors, such as pH and RNase activity, may affect the stability of the HTNV RNA in the urine, hindering the acquisition of the whole-genome sequence. Collection of urine specimens should be considered a pre-analytical factor that influences the stability of HTNV RNA in urine. Further investigation of handling urine specimens that optimizes the recovery of HTNV RNA is needed for clinical and molecular diagnosis of HFRS.

Reassortment and recombination are molecular mechanisms that confer genetic diversity in RNA viruses in nature [11]. Reassortment of SNV has more commonly been described in M segments than in L or S segments in nature and *in vitro* [33]. The less common reassortants of L or S segments than the M segment might be associated with the function of these viral proteins. The maintenance of L and S segments might be beneficial for replication, transcription, and assembly, resulting in the production of progeny possessing appropriate viral fitness. However, given our limited understanding of this genetic mechanism, the existence of reassortants that contain different combinations of segments should not be ignored. Infection with Guaroa virus generated the L segment reassortant, whereas S segment reassortant was observed between Bunyamwera and California encephalitis viruses [34]. In this study, the reassortment analysis demonstrated that HTNV of ROKA16-10 urine and serum in Pocheon-si showed a heterogeneous L segment but homogeneous M and S segments. Since the reassortant was not identified in the *A*. *agrarius* caught in Pocheon-si, it is necessary to confirm the presence of reassortant through targeted animal trapping. The continued epidemiology and large-scale surveillance of orthohantavirus remain to be investigated.

There are limitations to this study: 1) low coverage rate in urine specimens, 2) isolation of infectious particles in the urine, and 3) a small cohort of patients. The urine sampling date of ROKA16-10 was three days after onset. The urine sampling date of ROKA16-12, ROKA17-2, and ROKA17-15 was about 7 days after onset. The whole-genome sequences of HTNV ROKA16-10 were completely recovered, whereas the HTNV ROKA16-12, ROKA17-2, and ROKA17-15 showed low coverages and depth of viral reads using the multiplex PCR-based NGS. Whether the clinical manifestation of patients with HFRS affects the stability of urinary HTNV RNA remains to be determined. To examine the risk assessment of urinary HTNV, the isolation of infectious virions is needed from the urine specimens of patients with HFRS. In addition, whether the urine specimens are available for HTNV genomic detection and diagnosis of patients with HFRS in large cohorts awaits further investigation.

In conclusion, urinary genome sequences of HTNV were acquired by multiplex PCR-based NGS. Whole or partial genome sequences of HTNV from the urine enabled to track the putative infection sites of patients with HFRS by phylogeographically linking to the zoonotic HTNV from the reservoir host captured at endemic regions. In addition, phylogenetic pattern of the HTNV L segment showed a genetic configuration compatible with the genetic reassortment. This study provides insights into non-invasive diagnostics and genomic epidemiology of urinary viral genome sequencing against emerging HTNV outbreaks. It raises awareness among physicians regarding the presence of HTNV in the urine of patients with HFRS.

## Material and methods

### Ethics statement

Each patient sample was collected under informed written consent (AFCH18-IRB-004) of Armed Forces Medical Command (AFMC) of Republic of Korea (ROK) with approval for all aspects of human participants and case studies.

### Indirect immunofluorescence antibody test (IFAT)

Urine and serum were diluted 1:32 in phosphate-buffered saline (PBS), placed into wells of acetone-fixed Vero E6 cells infected with HTNV, and then incubated at 37°C for 30 min. After the wells were washed with PBS and distilled water (DW), fluorescein isothiocyanate-conjugated goat antibody to human immunoglobulin G (IgG) (ICN Pharmaceuticals, Laval, Canada) was added to the wells. The wells were incubated at 37°C for 30 min and washed with PBS and DW. The hantavirus-specific spot was examined under a fluorescence microscope (Axio Scope, Zeiss, Berlin, Germany).

### RNA extraction and reverse transcription PCR (RT-PCR)

Total RNA was extracted from specimens (urine and serum) using TRIzol LS Reagent (AMBION Inc., Austin, TX, USA). cDNA was synthesized using a High-Capacity RNA-to-cDNA kit (Applied Biosystems, Foster City, CA, USA) with random hexamer and OSM55 (5’-TAGTAGTAGACTCC-3’). Nested PCR was performed in 25 μL reactions containing 200 μM dNTP (Elpis Biotech, Daejeon, Korea), 0.25 U of SuperTherm Taq polymerase (JMR Holdings, London, UK), cDNA, and HTNV-specific primer sets [35]. Initial denaturation was performed at 95°C for 5 min, followed by 6 cycles of denaturation at 95°C for 40 s, annealing at 37°C for 40 s, elongation at 72°C for 1 min, followed by 32 cycles of denaturation at 95°C for 40 s, annealing at 42°C for 40 s, elongation at 72°C for 1 min, and then a final elongation at 72°C for 7 min using the ProFlex PCR System (Life technology, Carlsbad, USA).

### Quantitative real-time PCR (qPCR)

qPCR was performed using primers targeting the HTNV S segment with SYBR Green PCR Master mix (Applied Biosystems) on a Quantstudio 5 Flex Real-Time PCR System (Applied Biosystems). The cycling condition and primer information were previously described [35].

### Multiplex PCR-based next-generation sequencing (NGS)

Multiplex PCR was performed as previously described [35]. Multiplex PCR products were prepared using a TruSeq Nano DNA LT sample preparation kit (Illumina, San Diego, CA, USA) according to the manufacturer’s instructions. The samples were mechanically sheared using an M220 focused ultrasonicator (Covaris, Woburn, MA, USA). The cDNA amplicon was size-selected, A-tailed, ligated with indexes and adaptors, and enriched. The libraries were sequenced using a MiSeq benchtop sequencer (Illumina) with 2 × 150 bp with a MiSeq reagent V2 (Illumina).

### Phylogenetic analysis

The genome sequence of HTNV from sera of patients with HFRS and urine was aligned using the Clustal W method with the Lasergene program, version 5 (DNASTAR). Phylogenetic analysis was conducted using the maximum likelihood (ML) method in MEGA 7.0 [36]. Topologies were evaluated by bootstrap analysis of 1,000 iterations. In addition, MrBayes 3.2.7a program was used for a Bayesian analysis. Markov chain Monte Carlo (MCMC) runs with 6 chains of 10,000,000 generations were sampled every 1,000 generations (sumt burn-in of 25%). Maximum clade credibility trees were prepared in FigTree version 1.4.0.

### Analyses of genomic recombination and reassortment

Alignments of the concatenated HTNV L, M, and S segments were analyzed using the RDP4 package. Recombination/reassortment events were significantly suggested if two criteria were satisfied: the *p*-value (p) was under 0.05, and the RDPRCS was over 0.6. Recombination/reassortment events were considered possible when p was less than 0.05 and the RDPRCS was between 0.4 and 0.6. The likelihood of recombination/reassortment events was considered insignificant when the RDPRCS was under 0.4 with p<0.05. Reassortment events were analyzed using the GiRaF. Nucleotide alignments of HTNV tripartite genome segments were used as an input for MrBayes [37]. As input for the software, 1,000 unrooted candidate trees were estimated using the GTR+G+I substitution model, a burn-in of 50,000 iterations (25%) and sampling every 200 iterations. These trees were used to simulate the phylogenetic uncertainty of the segments. The default value of the confidence threshold was 0.7 for the data set; all events were at over 0.9 confidence levels [38]. The process was repeated ten times with ten independent MrBayes-based tree data per segment.
